# First experience in employing a complex digital support system accompanied by personal assistance to improve aftercare in patients with stroke or transient ischemic attack – results of the PostStroke-Manager feasibility study

**DOI:** 10.1038/s41598-025-89044-7

**Published:** 2025-02-17

**Authors:** Dominik Michalski, Joseph Classen, Daniela Geisler, Daniela Urban, Max Schreiber, Jean-Baptiste Tylcz, Richard Schmidt, Alexander Prost, Till Handel, Andreas Schneider, Stefan Lippmann, Markus Bleckwenn, Galina Ivanova

**Affiliations:** 1 Department of Neurology, University of Leipzig, Liebigstr. 20, 04103 Leipzig, Germany; 2 Innovation Center Computer Assisted Surgery, University of Leipzig, Semmelweisstr. 14, 04103 Leipzig, Germany; 3 Institute of General Practice, University of Leipzig, Ph.-Rosenthal-Str. 55, 04103 Leipzig, Germany; 4 Center for Psychosocial Medicine, University Medical Center Hamburg-Eppendorf, Martinistr. 52, 20246 Hamburg, Germany

**Keywords:** Stroke, Stroke aftercare, Secondary prevention, Digital support, App, Neurology, Stroke

## Abstract

Stroke aftercare is widely acknowledged as crucial yet challenging. Digital tools offer a promising strategy to improve aftercare and, thus, patients’ quality of life. However, it has not yet been investigated whether digital tools addressing different aspects of aftercare at the same time can be utilized in stroke patients. This study was intended to gain first experience in employing a complex patient-centered digital support system (i.e., the PostStroke-Manager) in individuals with stroke or transient ischemic attack (TIA) and thus examined its feasibility accompanied by personal assistance. This cohort study was carried out to enroll patients with stroke or TIA. Digital support was realized through an application installed on a tablet, a smartwatch, and a blood pressure monitor. Trained nurses, referred to as stroke pilots, provided personal assistance. After 6 or 12 months, patients were asked to evaluate the concept with digital and personal support as a whole and concerning single components of the program for individually experienced beneficial effects. Additional descriptive analyses comprised temporal courses of stroke-related impairments, quality of life, anxiety, depression, adherence, empowerment, blood pressure, LDL cholesterol, and HbA1c levels. Between January 2022 and December 2022, 815 patients were screened, and 43 were included in the study, of whom 36 completed follow-up assessments. The study met its predefined feasibility criterion, with at least 50% of program components receiving positive ratings from at least 75% of patients. The digital system as a whole (77.8%), personal assistance (75.0%), and interactions with stroke pilots (77.8%) received particularly positive ratings. Additionally, aspects such as vital sign monitoring, platforms for communication and medical records were rated positively. Longitudinal analyses revealed improvements in patients’ impairments, blood pressure, and LDL cholesterol levels, while most psychometric measurements remained stable. These findings indicate the feasibility of employing complex digital support systems for patient-centered aftercare in selected individuals with stroke or TIA. The challenge remains in extending benefits to those patients with more severe neurological impairments, highlighting the necessity for ongoing advancement in this field.

## Background

Stroke is a major vascular disease with about 94 million cases worldwide in 2021^1^. Estimations for Europe indicate an increase in the number of people living with stroke of about 27% until the year 2047^2^. Recent treatment of stroke includes, among others, specialized inpatient units and techniques targeting on the revascularization of brain-supplying vessels, which have demonstrated to reduce mortality and lesser functional impairment^3^. However, observations have indicated that 39% of patients exhibit persisting disabilities in the long term^4^.

Aftercare is thus acknowledged as crucial in stroke care, highlighted by initiatives such as the ‘Stroke Action Plan for Europe’ (SAP-E) with its domain ‘life after stroke’^5^. These initiatives also cover patients with a transient ischemic attack (TIA) as a further type of cerebral ischemia that is characterized by having the same risk factors and a remarkable risk for secondary events, respectively^4^. Aftercare aims to prevent secondary cerebrovascular events by, among others, optimal treatment of arterial hypertension, atrial fibrillation, and hyperlipidemia^6^. However, a more comprehensive perspective of aftercare also includes the detection of comorbidities such as anxiety, depression, and cognitive impairment, the best possible treatment of stroke-related disabilities (e.g., limb paresis, aphasia), and diverse support regarding, for instance, communication and mobility^5^. Another objective is to inform patients about cerebrovascular events and related risk factors to enhance adherence and empowerment. Along with initiatives focusing on a more patient-centered perspective^5^, patient-reported outcome measures (PROMs), e.g., in terms of health-related quality of life, have gained increasing attention when describing individual courses in the long term^7^.

Generally, the realization of stroke aftercare depends on individual efforts and the supportive infrastructure of the healthcare system. National projects for optimized aftercare in individuals with stroke and TIA investigated patient-centered interventions for up to two years^8,9^. They found beneficial effects regarding, for instance, blood pressure and control of hyperlipidemia, but not a reduction in secondary cerebrovascular events. Another approach that is currently under investigation focuses on the implementation of stroke pilots as specially trained nurses or other healthcare professionals who provide individual guidance within the healthcare infrastructure and diverse support to patients after stroke^10^. Still, existing challenges include the complexity of aftercare with naturally varying objectives depending on individual and potential barriers when supplying information among healthcare providers as well as between them and the patients themselves.

Digital tools may represent a promising strategy to, for instance, optimize secondary prevention, explore individual needs, assess PROMs, and allow different ways of communication with the overall goal of improving aftercare and, thus, patients’ quality of life following stroke or TIA^11–14^. Such tools may further optimize processes within the healthcare system, which may benefit patients and healthcare professionals. For example, such tools could help choose individual treatments based on updated patient data and save time by reducing expenditures, such as regarding documentation. In this light, the implementation of digital support systems for providing information and assistance was defined as a target for the year 2030 in the SAP-E^5^. Against this background, the PostStroke-Manager was developed as a complex patient-centered digital support system addressing various aspects with high priority in stroke aftercare at the same time (details below)^15^. The system included an application (app) with individual data and health records installed on a tablet with SIM card, connected with a blood pressure monitor and a smartwatch via Bluetooth. These mobile devices were connected to a server, enabling the exchange of information and direct communication to stroke pilots at the initially treating hospital and the general practitioner, who had access to data via a web portal.

However, it has not yet been investigated whether digital tools addressing several aspects of aftercare at the same time can be employed in patients with cerebrovascular events and thus be used to improve individual aftercare. To gain first experience in using a complex patient-centered digital support system in individuals with stroke or TIA, this study evaluated the feasibility of the PostStroke-Manager concept accompanied by personal assistance through stroke pilots. Assessment of feasibility was based on patients’ ratings, while digital and personal support as a whole and specific components of the program were rated individually regarding the experienced beneficial effect. In addition to an overall evaluation, this study aimed to provide details on which aspects of aftercare are eligible to be supported digitally. Further descriptive analyses comprised temporal courses of patients’ stroke-related impairments, health-related quality of life, anxiety, depression, medical adherence, and empowerment, reflecting a potentially additional feature of the system to record and describe relevant determinants in stroke aftercare, which could help tailor aftercare more individually.

## Methods

### Study design and patients

For this observational cohort study, patients treated between January 2022 and December 2022 at the Stroke Unit and Neurological Intensive Care Unit of the Department of Neurology, University of Leipzig, Germany, were screened. An observation period of 12 months was initially planned for all patients, whereby the duration of the study within the scope of the underlying project was limited from January 2022 to June 2023. Due to the low recruitment, which was significantly influenced by the SARS-CoV2 pandemic, the protocol was amended by adding a group of patients with an observation period of 6 months, allowing an extension of the recruitment period by 6 months.

Patients were considered for participation if they had an ischemic or hemorrhagic stroke or a transient ischemic attack (TIA). According to existing conventions^16^, stroke was defined by persisting or transient neurological symptoms with an ischemic lesion or hemorrhage on computed tomography or magnetic resonance imaging, whereas TIA was defined as transient neurological symptoms without radiological evidence of an ischemic lesion or hemorrhage. The main inclusion criteria also comprised an age of at least 18 years, no or moderate functional impairment before the cerebrovascular event that qualified for study participation [pre-(modified Rankin Scale (mRS))^17^ 0–2], stable course of symptoms during the hospital stay, allowing hospital discharge, and residence in or around the city of Leipzig, Germany. As the involvement of physicians who will treat patients after hospital discharge according to standards in stroke aftercare was essential, the inclusion of patients also required the consent of their general practitioners to support the study.

Main exclusion criteria were an inability to use the digital system or mobile devices (e.g., severe aphasia or neglect and hemiplegia), physical impairment impeding the realization of the observation period (e.g., cancer with a relevant reduction of life expectancy), clinically relevant psychiatric disorders, and known dementia.

Due to the observational character of this study, both digital support and personal assistance through stroke pilots should not have negatively influenced the standard care of patients, primarily managed by general practitioners after discharge. However, based on communications between patients, stroke pilots, and the general practitioner, relevant information that had emerged during the observation period within the study, for example, suspected emotional comorbidities, was considered for individual treatment.

The study was approved by the local ethics committee at the Medical Faculty of the University of Leipzig, Germany (reference number 504/20-ek). Further, the study was pre-registered in the German Registry for Clinical Trials (DRKS00023213), and a detailed description of the study had already been published^15^.

All patients provided written informed consent. Study procedures were performed in accordance with the relevant guidelines and regulations. Reporting of results considered the STROBE criteria for cohort studies^18^.

### Digital support for patients

A set of devices including a tablet (Galaxy Tab S6lite, Samsung, Schwalbach, Germany) equipped with a SIM card for universal internet access and with the PostStroke-Manager app installed, two smartwatches (TicWatch Pro 2020, Mobvoi, Hong Kong, China), and a blood pressure monitor with ECG function (Veroval 2in1, Hartmann, Heidenheim, Germany) was used to realize digital support for patients. The set of devices was handed over to study participants during their initial hospital stay. Upon initiation, the PostStroke-Manager app, which was newly developed within the underlying project, was configured and enriched with individual patient data, and from now on allowed each patient to use 10 components, including (1) storage of individual data (e.g., individual risk factors, neurological symptoms and impairments related to the qualifying event), (2) platform for medical records, (3) platform for communication with stroke pilots and general practitioner (text messages and video telephony), (4) calendar, e.g., for individual appointments, (5) psychometric measurements using standardized questionnaires (see below), (6) medication schedule, (7) recording of blood pressure and ECG signals, (8) platform for managing individual goals and actions, depending on individual risk factors and impairments, (9) neuropsychological exercises, and (10) information portal, including, e.g., pathophysiological details and risk factors of the underlying event and related treatment option. Patients also had access to a database with frequently asked questions (FAQs) and an electronic ticket system for solving technical problems.

### Study procedure

After an initial explanation and training phase during the hospital stay, patients were discharged and used the digital system for 6 or 12 months, accompanied by personal assistance from two stroke pilots. These pilots were experienced nurses specially trained in case management (acquired at the Dresden International University, Dresden, Germany) and additional training on stroke-related issues according to the educational program of the German Stroke Foundation (Gütersloh, Germany). The Stroke pilots helped patients navigate through the rehabilitation process and the outpatient setting. They provided diverse support to patients, e.g., by cooperatively developing a biopsychosocial disease model based on individual risk factors, checking medication adherence, and evaluating individual disabilities and comorbidities with associated needs and goals. The two pilots also assisted patients using the digital system and managed technical issues together with the group members who developed the technological part of the PostStroke-Manager system.

With standardized questionnaires integrated into the patients’ app and predefined intervals, the digital system allowed self-assessment of health-related quality of life [short form (36) health survey (SF-36)^19^, 3-month intervals), anxiety and depression [hospital anxiety and depression scale (HADS)^20^, 3-month intervals], adherence [adherence assessment questionnaire (AAQ)^21^, and adherence barriers questionnaire (ABQ)^22^, 6-month interval for each], and empowerment [healthcare empowerment inventory (HCEI)^23^, 3-month interval].

Clinical visits were performed at intervals of 3 months by a study physician and one stroke pilot throughout the observation period of either 6 or 12 months. These visits included, among others, assessment of parameters describing the degree of neurological symptoms, functional, and cognitive impairment [National Institutes of Health Stroke Scale (NIHSS)^24^, mRS^17^, Barthel index^25^, mini mental status examination (MMSE)^26^. Visits were also used to explore the experienced burden of patients when using the digital support system, assessed by a 4-point scale including ‘no burden’, ‘low burden’, ‘relevant burden’, and ‘severe burden’. As a predefined study action, patients reporting a ‘relevant burden’ would be asked to either stop or continue the study, while a ‘severe burden’ would have resulted in a stop of participation.

Data were usually entered digitally via the patient’s app or the web portal used by the stroke pilots. An additional web portal was used by general practitioners who treated the patients after discharge, including, for instance, prescription of medication and laboratory tests regarding glucose and lipid metabolism. Available results from laboratory tests were transferred into the digital system by stroke pilots and general practitioners based on reports emerging from the initial assessment during the hospital stay and subsequent laboratory tests after discharge. Blood pressure and heart rate were acquired using the individual mobile blood pressure monitors connected with the patients’ apps.

Extensive monitoring of the device management, data quality, and process continuity was done by the group members who developed the technological part of the PostStroke-Manager system. Members of the technological team also educated and supported the stroke pilots in using the PostStroke-Manager system.

### Outcome measures

The primary outcome was the feasibility of the PostStroke-Manager concept and, thus, the employment of a complex patient-centered digital support system with personal assistance in patients with stroke and TIA. Addressing the patients’ perspective, a newly conceptualized questionnaire with 14 items in total was used to assess individually experienced benefits. Two questions covered the potential benefits of the digital system as a whole and personal assistance by stroke pilots. Nine questions assessed perspectives on each digital system component respectively (except psychometry). Three questions gave the opportunity to (1) rate the technical support, including a database with FAQs, (2) an electronic ticket system, and (3) personal contact with stroke pilots in case of technical issues. For each question, patients were able to choose one of the following answers: ‘very high benefit’, ‘high benefit’, ‘sufficient benefit’, ‘some benefit’, ‘rare benefit’, and ‘no benefit’. As defined by the study protocol, the PostStroke-Manager would have been evaluated as feasible if at least 50% of the questions were rated positive by at least 75% of patients^15^. According to protocol, a positive rating included ‘very high benefit’, ‘high benefit’, ‘sufficient benefit’, and ‘some benefit’. Additionally, patients were asked about their personal preference regarding support in stroke aftercare (optional answers: ‘surely pilots’, ‘rather pilots’, ‘both in a similar manner’, ‘rather app’, and ‘surely app’).

Secondary outcomes included feasibility depending on the patients’ cerebrovascular event that qualified for study participation, i.e., ischemic, hemorrhagic stroke, and TIA. Further, qualitative feedback from stroke pilots and general practitioners, and the burden experienced by patients when using the digital system served as secondary outcomes. In addition, descriptive analyses regarding the temporal course of stroke-related symptoms, impairments, health-related quality of life, anxiety, depression, cognitive function, medical adherence, and empowerment were conducted.

### Statistical analyses and figure generation

Analyses were performed with the SPSS software package (version 29.0; IBM Corp., Armonk, NY, USA). A descriptive data analysis was carried out, including proportions for categorical variables and medians with interquartile ranges (Q1–Q3) for continuous variables, considering the relatively small sample size and the non-normal distribution of several variables as evaluated by the Kolmogorov-Smirnov test. To compare values between baseline and 6 months as well as baseline and 12 months, a non-parametric test (i.e., the Wilcoxon test) was chosen. To reduce the family-wise error rate, the Bonferroni correction method was applied (*α*_Bonferroni_ < 0.05 / 44 tests), resulting in an adjusted alpha level of 0.001. Thus, statistical significance was assumed at a p-value < 0.001. Figures were created with R Statistical Software in R Studio (version 2023.09.0 + 463)^27,28^, Microsoft Excel, and PowerPoint for Mac (version 16.8, 2023, Microsoft, Redmond, USA).

## Results

### Recruitment and patients’ characteristics

**Fig. 1:**
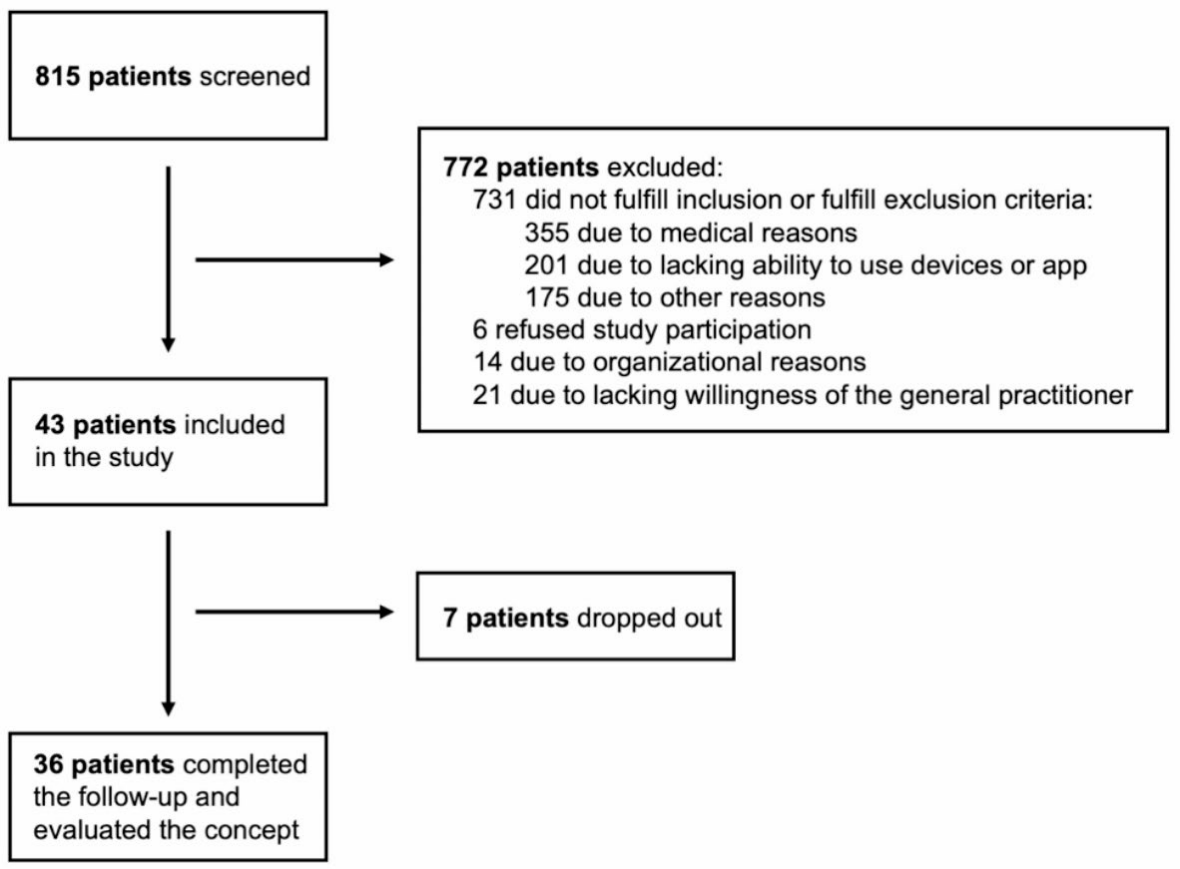
Flowchart with details regarding the number of screenings, study enrollments, and patients who completed the observation period of either 6 or 12 months.

During the one-year recruitment period, a total of 815 patients were screened. Forty-three patients entered the study, and 36 of them completed the follow-up of either 6 or 12 months (Fig. 1). According to the study protocol, most exclusions were due to medical reasons or lacking ability to use devices or an app, mainly caused by a relevant functional impairment before the qualifying event (pre-mRS > 2), severe clinical symptoms emerging from stroke or known dementia. Further exclusions resulted from circumstances such as a residency out of the area where the study was conducted and a short-term hospital stay not allowing extensive study procedures. Remarkably, 21 patients were eligible for the study but could not be included due to missing support from the general practitioner. Dropouts occurred after predominantly short observational periods: 6 patients within about 4 weeks and one patient after about 25 weeks.

Overall, this study was based on the evaluation of 36 patients, of whom 14 had a follow-up of 6 months and 22 of 12 months. The cohort comprised 31 patients with ischemic stroke and 5 with TIA, while no cases with hemorrhagic stroke were enrolled in the study. The baseline characteristics of patients and information on the acute treatment performed and the course during hospital stay are given in Table 1.

**Table 1:** Patients‘ characteristics.

Variable	
Age in years (M (IQR))	66.0 (57.3–73.3)
Sex (female, n (%))	13 (36.1)
Risk factors Body mass index (M (IQR)) Arterial hypertension (n (%)) Dyslipidemia / prior use of statins (n (%)) Diabetes mellitus (n (%)) Nicotine consumption (n (%)) Atrial fibrillation (n (%))	26.9 (24.8–29.4) 32 (88.9) 36 (100.0) 8 (22.2) 6 (16.7) 3 (8.3)
Pre-mRS (M (IQR))	0.0 (0.0–0.0)
Acute treatment No recanalization approach (n (%)) IVT (n (%)) EVT with or without IVT (n (%))	25 (69.4) 7 (19.4) 4 (11.1)
mRS at admission (M (IQR))	2.0 (2.0–3.8)
mRS at discharge (M (IQR))	2.0 (2.0–2.0)
NIHSS at admission (M (IQR))	2.0 (1.0–4.8)
NIHSS at discharge (M (IQR))	0.0 (0.0–1.0)
Hospital stay in days (M (IQR))	5.5 (4.0–7.0)

Information relates to 36 patients. M: median, IQR: interquartile range, n: number of patients, mRS: modified Rankin Scale, NIHSS: National Institutes of Health Stroke Scale, IVT: intravenous thrombolysis, EVT: endovascular treatment (i.e., mechanical recanalization).

### Evaluation of the complex digital support system and personal assistance

**Fig. 2:**
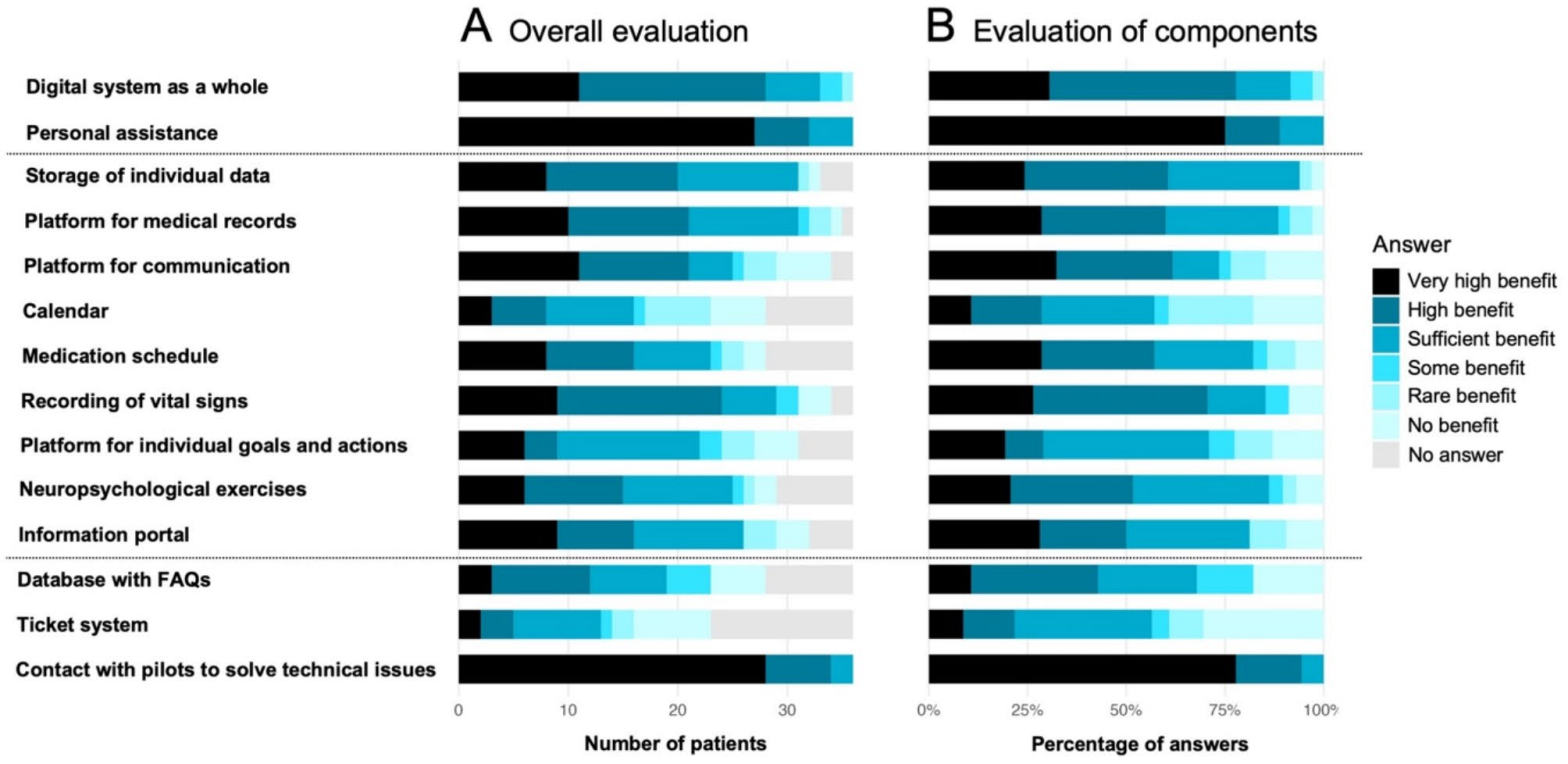
Distribution of patients’ ratings regarding 14 components of the PostStroke-Manager concept. (**A**) Overall evaluation with numbers of patients rating the concept with different levels of experienced benefit, including those with missing answers. (**B**) Evaluation considering only existing answers. FAQs: frequently asked questions.

After participation for 6 or 12 months, most patients rated the PostStroke-Manager concept in a beneficial way, especially the digital system as a whole, the personal assistance, and the contact with stroke pilots to solve technical issues (Fig. 2A). However, some components were not rated by all participants, usually because some patients did not use all available components and therefore refrained from rating them. In consequence, missing values were not considered for the evaluation regarding the most beneficial components (Fig. 2B). Components primarily rated with ‘very high benefit’ were the personal assistance (27/36, 75.0%) and the contact with pilots to solve technical issues (28/36, 77.8%). The following components were largely rated with ‘very high benefit’ or ‘high benefit’: the digital system as a whole (28/36, 77.8%), the recording of vital signs (24/34, 70.6%), the platform for communication (21/34, 61.8%), the storage of individual data (20/33, 60.6%), the platform for medical records (21/35, 60.0%), the medication schedule (16/28, 57.1%), the neuropsychological exercises (15/29, 51.7%), the information portal (16/32, 50.0%), and the database with FAQs (12/28, 42.9%). In contrast, the calendar (8/28, 28.6%) and the ticket system (5/23, 21.7%) were less frequently rated with ‘very high benefit’ or ‘high benefit’.

In consequence, the primary endpoint of this study (a rating of at least 50% of the questions with ‘some benefit’ or better by at least 75% of participants) was met. In detail, 7 out of 14 questions were evaluated positively by 31/36 (86.1%) patients including missing answers, and 34/36 (94.4%) rated at least 50% of questions positively. Regarding their preferred type of future support in stroke aftercare (either digitally or personally), 21/36 patients (58.3%) answered ‘both in a similar manner’, 1/26 (2.8%) ‘rather app’, 5/36 (13.9%) ‘rather pilots’, and 9/36 (25.0%) ‘surely pilots’. The planned sub-analysis regarding the cerebrovascular event that qualified for study participation was waived since the study cohort included predominantly patients with ischemic stroke and a few with TIA but not hemorrhagic stroke, and thus, the intended sample size of 30 patients in each subgroup was not realized.

During the observation period of either 6 or 12 months, most patients experienced no or only low burden emerging from the digital system. In detail, at 3 months, 16/36 (44.4%) reported ‘no burden‘, 17/36 (47.2%) ‘low burden‘, 3/36 (8.3%) ‘relevant burden‘, and no one (0%) ‘severe burden‘. At 6 months, 14/36 (38.9%) patients reported ‘no burden‘, 21/36 (58.3%) ‘low burden‘, 1/36 (2.8%) ‘relevant burden‘, and no one (0%) ‘severe burden‘. At 9 months, 13/22 (59.1%) patients experienced ‘no burden‘, 8/22 (36.4%) ‘low burden‘, 1/22 (4.5%) ‘relevant burden‘, and no one (0%) ‘severe burden‘. At 12 months, 9/22 (40.9%) experienced ‘no burden‘, 13/22 (59.1%) a ‘low burden‘, while no one experienced a ‘relevant‘ or a ‘severe burden‘ (0% each). All patients who had reported a ‘relevant burden’ during the study period agreed to continue study participation.

Among the unstructured feedback from the two stroke pilots, positive aspects included, for instance, (1) the permanent availability of patient-related data such as the medication plan and medical records, simplifying the daily work of pilots, (2) the simple way to stay in contact with patients by text messages, and (3) effortless knowledge transfer to patients by using parts of the information portal. Challenging aspects exemplarily included (1) the need for mobile data services (wireless network or telephone network, the latter requirement for SIM card use) and battery power when using mobile devices with above-average intensity, (2) the high rate of contacts regarding technical issues in this experimental stage, and (3) time-consuming instruction of patients in the use of hard- and software. Unstructured feedback was also given from general practitioners participating in the study, whereby positive aspects included, for instance, (1) the ability to store medical records in the digital system, (2) the communication with stroke pilots, and (3) an increased patients’ awareness for active participation and appointments. Challenging aspects exemplarily included (1) technical issues during login or upload of records via the experimental general practitioners’ web portal, (2) a lacking added value on the individual level in patients without impairments in daily living, and (3) differences in blood pressure measurements compared to external recordings, which was unanticipated.

### Temporal courses of individual symptoms, functional impairments, emotional comorbidities, attitudes, vital signs, and laboratory results

**Fig. 3:**
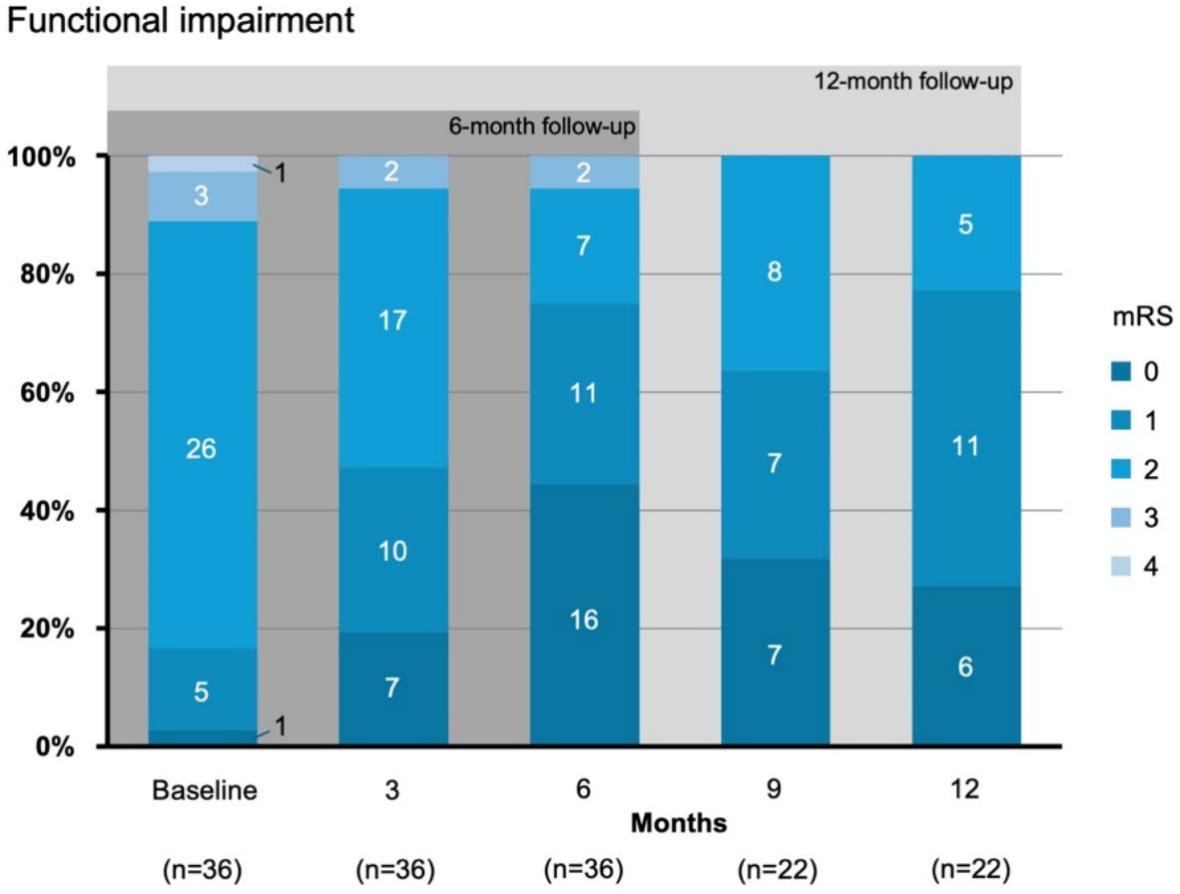
Functional impairment, assessed by the modified Rankin Scale (mRS) during follow-up of either 6 or 12 months, shown in intervals of 3 months with inserted numbers of patients and respective proportions. n: number of patients.

In terms of neurological symptoms, patients had a relatively low NIHSS (about 1) at baseline, which declined significantly during the next 6 months (Table 2). A similar trend was observed for functional impairment, which was assessed by the mRS, starting with a relatively low value at baseline that declined significantly towards 6 and 12 months. When focusing on the proportion of patients with no or irrelevant impairment (mRS 0 to 1), an increase was observed during the 6- and 12-month observation period (Fig. 3). The Barthel index was found to be stable with relatively high values, indicating little or no need for support in daily living. In terms of cognitive impairment, patients started with relatively high scores on the MMSE, indicating nor or discrete impairment, with no significant change over the following 6 or 12 months (Table 2).

**Table 2:** Course of stroke-related symptoms and impairments.

Variable	Baseline	3 months	6 months	9 months	12 months	*p* *	*p* ^#^
NIHSS (M (IQR) [n])	1.0 (0.0–1.0) [36]	0.0 (0.0–1.0) [36]	0.0 (0.0–0.0) [36]	0.0 (0.0–1.0) [22]	0.0 (0.0–1.0) [22]	< 0.001	n.s.
mRS (M (IQR) [n])	2.0 (2.0–2.0) [36]	2.0 (1.0–2.0) [36]	1.0 (0.0–1.75) [36]	1.0 (0.0–2.0) [22]	1.0 (0.0–1.25) [22]	< 0.001	< 0.001
Barthel index (M (IQR) [n])	100.0 (100.0–100.0) [36]	100.0 (100.0–100.0) [36]	100.0 (100.0–100.0) [36]	100.0 (100.0–100.0) [22]	100.0 (100.0–100.0) [22]	n.s.	n.s.
MMSE (M (IQR) SD [n])	29.0 (27.0–29.8) [36]	29.0 (28.8–30.0) [34]	30.0 (29.0–30.0) [35]	29.0 (29.0–30.0) [22]	30.0 (28.8–30.0) [22]	n.s.	n.s.

Data based on standardized assessments, i.e., NIHSS: National Institutes of Stroke Scale, mRS: modified Rankin Scale, Barthel index, and MMSE: mini mental state status examination, M: median, IQR: interquartile range, n: number of patients, p-values: *: baseline to 6 months, ^#^: baseline to 12 months, n.s.: non-significant.

With regard to health-related quality of life, the patients initially showed relatively high values for most of the 8 subscales of the SF-36 used, which indicates a predominantly good health-related quality of life (Table 3). Overall, stable values were found during observation periods of 6 and 12 months, respectively. The anxiety and depression levels, which were assessed using the HADS, were also low at baseline and during the observation periods of up to 12 months, indicating a low emotional burden overall (Table 3).

**Table 3:** Course of health-related quality of life, anxiety, and depression.

Variable	Baseline	3 months	6 months	9 months	12 months	*p* *	*p* ^#^
Physical functioning (M (IQR) [n])	70.0 (41.3–95.0) [36]	85.0 (55.0–96.3) [34]	85.0 (55.0–95.0) [35]	70.0 (38.8–95.0) [22]	75.0 (30.0–92.5) [21]	n.s.	n.s.
Physical role (M (IQR) [n])	62.5 (0.0–100.0) [36]	50.0 (25.0–100.0) [34]	50.0 (0.0–100.0) [35]	12.5 (0.0–100.0) [22]	50.0 (0.0–100.0) [21]	n.s.	n.s.
Emotional role (M (IQR) [n])	83.3 (33.3–100.0) [36]	66.7 (0.0–100.0) [34]	100.0 (0.0–100.0) [35]	50.0 (0.0–100.0) [22]	66.7 (0.0–100.0) [21]	n.s.	n.s.
Vitality (M (IQR) [n])	60.0 (35.0–78.8) [36]	60.0 (40.0–75.0) [34]	65.0 (45.0–80.0) [35]	55.0 (33.8–70.0) [22]	55.0 (40.0–72.5) [21]	n.s.	n.s.
Mental health (M (IQR) [n])	74.0 (65.0–84.0) [36]	80.0 (64.0–85.0) [34]	80.0 (56.0–92.0) [35]	72.0 (59.0–85.0) [22]	68.0 (60.0–88.0) [21]	n.s.	n.s.
Social functioning (M (IQR) [n])	87.5 (65.6–100.0) [36]	93.8 (62.5–100.0) [34]	100.0 (75.0–100.0) [35]	75.0 (50.0–100.0) [22]	87.5 (62.5–100.0) [21]	n.s.	n.s.
Pain (M (IQR) [n])	78.8 (57.5–100.0) [36]	85.0 (57.5–100.0) [34]	77.5 (57.5–100.0) [35]	82.5 (41.9–100.0) [22]	67.5 (50.0–100.0) [21]	n.s.	n.s.
General health (M (IQR) [n])	60.0 (46.3–65.0) [36]	60.0 (52.5–76.3) [34]	55.0 (45.0–70.0) [35]	55.0 (38.8–65.0) [22]	55.0 (47.5–67.5) [21]	n.s.	n.s.
Anxiety (M (IQR) [n])	5.0 (3.0–9.0) [35]	4.0 (2.0–8.0) [35]	4.0 (2.0–8.0) [35]	5.5 (2.0–9.3) [22]	5.0 (3.0–8.0) [21]	n.s.	n.s.
Depression (M (IQR) [n])	2.0 (1.0–6.0) [35]	3.0 (1.0–6.0) [35]	3.0 (1.0–7.0) [35]	5.5 (2.8–8.0) [22]	4.0 (2.0–7.5) [21]	n.s.	n.s.

Data based on standardized questionnaires, i.e., SF-36 for health-related quality of life, and HADS for anxiety and depression. M: median, IQR: interquartile range, n: number of patients, p-values: *: baseline to 6 months, ^**#**^: baseline to 12 months, n.s.: non-significant.

For adherence and associated barriers (e.g., individual, related to medication and the healthcare system), predominantly stable values were found during the observation periods of either 6 or 12 months (Table 4). However, a significant decrease and thus a positive course was seen for treatment adherence from the initial value to 6 months. With regard to individual empowerment, i.e., commitment in terms of information, engagement, cooperation, and tolerance of uncertainty, the patients showed relatively high values at baseline, which were also maintained throughout the study (Table 4).

**Table 4:** Course of adherence and empowerment.

Variable	Baseline	3 months	6 months	9 months	12 months	*p* *	*p* ^#^
Adherence (M (IQR) [n])	12.0 (10.0–14.0) [36]		10.0 (9.0–12.0) [35]		10.0 (9.0–12.0) [21]	< 0.001	n.s.
Adherence barriers (M (IQR) [n])	26.5 (24.0–29.8) [36]		25.0 (21.0–31.0) [35]		26.0 (23.5–29.0) [21]	n.s.	n.s.
Engagement (M (IQR) [n])	16.0 (14.0–17.0) [36]	16.0 (15.8–18.0) [34]	16.0 (15.0–18.0) [35]	16.0 (15.8–17.3) [22]	16.0 (14.5–17.0) [21]	n.s.	n.s.
Tolerance of uncertainty (M (IQR) [n])	16 (13.3–17.0) [36]	15.5 (13.8–16.3) [34]	16.0 (14.0–16.0) [35]	16.0 (15.0–17.0) [22]	16.0 (14.0–17.0) [21]	n.s.	n.s.

Data based on standardized questionnaires, i.e., AAQ and ABQ for adherence and adherence barriers, and HCEI for engagement and tolerance of uncertainty. M: median, IQR: interquartile range, n: number of patients, p-values: *: baseline to 6 months, ^#^: baseline to 12 months, n.s.: non-significant.

With reference to the assessments at baseline, systolic and diastolic blood pressure significantly decreased towards 6 months (Table 5). For laboratory results, a significant decrease from baseline to the last available time point was observed for LDL cholesterol but not for HbA1c (Table 6).

**Table 5:** Course of vital signs.

Variable	Baseline	3 months	6 months	9 months	12 months	*p* *	*p* ^#^
Systolic blood pressure (M (IQR) [n])	148.0 (128.8–167.3) [34]	128.0 (114.0– 132.5) [34]	125.0 (117.5–133.5) [33]	122.0 (115.0–132.0) [21]	125.0 (113.0–147.0) [21]	< 0.001	n.s.
Diastolic blood pressure (M (IQR) [n])	84.5 (75.3–94.3) [34]	76.0 (67.5–81.3) [34]	74.0 (68.0–80.5) [33]	73.0 (66.5–83.0) [21]	77.0 (67.5–89.0) [21]	< 0.001	n.s.
Heart rate (M (IQR) [n])	74.5 (67.0–87.3) [34]	70.5 (63.5–84.0) [34]	73.0 (62.0–82.0) [33]	72.0 (67.0–78.5) [21]	69.0 (62.5–87.0) [21]	n.s.	n.s.

M: median, IQR: interquartile range, n: number of patients, p-values: *: baseline to 6 months, ^#^: baseline to 12 months, n.s.: non-significant.

**Table 6:** Course of laboratory results.

Variable	Baseline	Last available	*p*
LDL cholesterol (M (IQR) [n])	3.4 (2.7–4.0) [36]	1.8 (1.4–2.3) [31]	< 0.001
HbA1c (M (IQR) [n])	5.7 (5.5–5.9) [35]	6.0 (5.6–6.5) [23]	n.s.

M: median, IQR: interquartile range, n: number of patients, n.s.: non-significant.

## Discussion

This study investigated the feasibility of the PostStroke-Manager concept to gain the first experience in employing a complex patient-centered digital support system with personal assistance in patients with stroke or TIA. Given the complexity of aftercare and numerous theoretical benefits arising from digital support systems^12,14^, the PostStroke-Manager was developed to address several highly prioritized aspects of aftercare at the same time, such as secondary prevention by an optimized recording of blood pressure, intersectoral communication, assessment of PROMs, individual adherence, and empowerment.

The study’s main finding is that complex digital tools can be employed in selected patients with stroke and TIA, as the predefined primary endpoint, which focused on feasibility, was met. In particular, the newly developed digital system as a whole, the personal support, and contact with stroke pilots for technical questions were rated positively, while digital components such as the recording of vital signs, communication as well as medical record platforms, and the storage of individual data, were rated as advantageous. At the same time, patients experienced a relatively low burden when using the system. These findings are essential for further development of digitally supported stroke aftercare. Since previous approaches have mainly focused on individual aspects, such as information about patients’ risk factors or an optimized blood pressure measurement^29,30^, the present result implies that future conceptualizations could consider more complex, standardized, or even individualized compositions. As a further important observation made in this study, most patients similarly preferred digital and personal support when asked for future assistance in aftercare.

This study also emphasizes the challenges of implementing a complex digital support system in stroke aftercare. Many persons had to be screened to realize an appropriately sized cohort of stroke and TIA patients using the PostStroke-Manager concept. One reason for this could be that the inclusion and exclusion criteria were rigorous, which was necessary for this first approach to implementing such a complex system. One further reason could be that the general practitioners’ support was required very early after the cerebrovascular event, i.e., a point in time when patients are naturally treated in a hospital setting. Consequently, the available cohort of patients was characterized by minor neurological symptoms and low functional impairment, as indicated by a low NIHSS and mRS at baseline. The screening process also indicates that substantial advancement is needed to enable most patients access to digitally-based aftercare solutions. Challenges include, for instance, poor premorbid conditions, severe clinical symptoms emerging from the cerebrovascular event, and cognitive impairment. At the same time, specific symptoms like aphasia or hemianopsia may also prevent using a complex digital support system. If people are unable to use digital systems after a stroke, involving relatives or caregivers could be an option to enable indirect use and, in some cases, positive effects.

In addition to the patient-centered evaluation with several implications for future directions, relevant aspects emerged from the unstructured feedback of participating stroke pilots and general practitioners. Recognized advantages included, among others, an improved availability of individual health records and different aspects of communication.

The second part of the study included descriptive analyses regarding the temporal course of patients’ characteristics, such as neurological symptoms, emotional comorbidities, and health-related quality of life, that are particularly interesting in stroke aftercare. With intervals of 3 months, a variety of assessments were realized, using, for instance, the mRS, NIHSS, Barthel index, MMSE, HADS, and SF-36. Regarding neurological symptoms and functional impairments, which started at an overall low level, a decrease was noticed during the observation period of 6 months and, at least in part, of 12 months, probably relating to a mixture of the natural course and specific rehabilitative actions. Health-related quality of life was found to be predominantly present at a relatively high level in participating patients without a relevant change during follow-ups up to 12 months. Emotional distress did not change significantly over time, as indicated by stable indicators of anxiety and depression. This is a remarkable observation, considering that another study reported a relevant burden in the long term after stroke^31^. For adherence, a positive trend was seen within the first 6 months of digital and personal assistance. However, this observation was not maintained towards 12 months, which might be related to a transient effect of suffering from stroke or TIA. Concerning empowerment, however, patients were characterized by relatively high values, reflecting their attitudes towards individual health promotions, which might also be the reason for participating in the study.

Regarding the blood pressure and heart rate, analyses of this study with intervals of 3 months indicated a decreasing course within a period of the first 6 months. The reduction of the systolic blood pressure to values below 140 mmHg is in good accordance with the recommendations for blood pressure management in secondary stroke prevention^6^. However, positive blood pressure modulation after stroke was also seen in patient-centered interventional programs without or with digital support^9,32^ and thus cannot be associated causally with the employed PostStroke-Manager concept. For laboratory parameters, HbA1c, as a value reflecting the glucose metabolism, was found stable over time, while a significant reduction of LDL cholesterol for about 1.4 mmol/L was observed. The achieved LDL cholesterol level, about 2 mmol/L, is almost following the recommendations of 1.8 mmol/L^6^. However, positive modulation of lipid metabolism was also observed in aftercare programs without digital support^8,9^. Thus, it cannot necessarily be linked to the applied concept of combining both digital and personal assistance.

The realized longitudinal analyses indicate that complex digital support systems can be used to record and describe various patients’ characteristics over time. This feature might help to tailor aftercare more individually while healthcare professionals could recognize unfavorable trends early, and interventions could be initiated promptly. From a more scientific perspective, analyses in larger cohorts would allow a more detailed exploration of post-acute care in the long term, e.g., regarding individual symptoms and PROMs, but also regarding the complexity of stroke aftercare.

This study has some limitations. First, the number of enrolled patients is small, and among cerebrovascular events, only ischemic stroke and TIA are considered, limiting the study’s conclusion to a specific group of individuals. Therefore, future initiatives are needed to evaluate complex digital support systems in larger cohorts of patients who experienced TIA, ischemic, or hemorrhagic stroke, including those with more severe impairment. Enrollment might also be enhanced when general practitioners are more involved in conceptual issues, and detailed information regarding digital approaches would be provided much earlier before participation. Second, in the absence of an already existing tool for assessing complex digital support systems in patients with cerebrovascular events, the feasibility evaluation was based on a newly conceptualized questionnaire. Patients rated specific components of the PostStroke-Manager concept concerning the individually experienced benefit using a scale with 6 options, while 4 were considered positive. Although the criterion for feasibility was chosen rigorously, the overall positive finding might be somewhat overinterpreted. Future studies should thus make use of more sophisticated assessments. Third, dropouts could not be considered for analyses as most of them occurred early during follow-up with the result that parameters describing the course of, for instance, clinical and psychometric features, are lacking. As this group of patients might be relevant when exploring factors that could promote discontinuing digitally-based support programs, future studies might focus on their characterization. Fourth, since the present study was intended to investigate a combined approach with digital and personal (i.e., face-to-face) support, no statement can be made about the feasibility of an exclusively digital support system. Given the theoretical benefit of digital tools to support aftercare in a patient-centered way, and, at the same time, save resources on the side of healthcare professionals, future initiatives should also include digital solutions that patients can handle without or with minor assistance. In addition to studies focusing on feasibility, subsequent trials are needed to address the efficacy of digital tools in stroke aftercare, particularly regarding secondary cerebrovascular events and patients’ health-related quality of life.

## Conclusions

This study, for the first time, indicates that complex patient-centered digital support systems with mobile and sensor-based technologies, such as the PostStroke-Manager, accompanied by personal assistance, can be employed in selected individuals with stroke or TIA. However, this observation is limited to a relatively small proportion of patients, including those with good premorbid conditions and no or minor neurological symptoms or functional impairments due to the cerebrovascular event. Advancements are thus needed to extend benefits emerging from digital support systems to patients with, for instance, more severe neurological impairments. In addition, the study indicated that the applied approach of using a digital support system in stroke aftercare could be used to record and describe patients’ characteristics, such as symptoms, impairments, and PROMs, which could help to tailor treatments more individually and allow further research regarding the complexity of post-acute care.

## Data Availability

Data underlying this study will be made available in a deidentified form upon reasonable request from a qualified investigator. Requests can be submitted to the corresponding author with information regarding the planned investigation and expected scientific value when adding data of this study, the institution in where the data will be processed, and the planned referencing to the PostStroke-Manager project and its researchers.

## Declarations

### Statement on use of AI and AI-assisted technologies

Grammarly (Grammarly Inc., San Francisco, CA, USA) was used to check and improve the manuscript’s language and readability without applying generative AI services.
